# Analysis of Postvaccination Breakthrough COVID-19 Infections Among Adults With HIV in the United States

**DOI:** 10.1001/jamanetworkopen.2022.15934

**Published:** 2022-06-07

**Authors:** Sally B. Coburn, Elizabeth Humes, Raynell Lang, Cameron Stewart, Brenna C. Hogan, Kelly A. Gebo, Sonia Napravnik, Jessie K. Edwards, Lindsay E. Browne, Lesley S. Park, Amy C. Justice, Kirsha S. Gordon, Michael A. Horberg, Julia M. Certa, Eric Watson, Celeena R. Jefferson, Michael J. Silverberg, Jacek Skarbinski, Wendy A. Leyden, Carolyn F. Williams, Keri N. Althoff

**Affiliations:** 1 Department of Epidemiology, Johns Hopkins Bloomberg School of Public Health, Baltimore, Maryland; 2 Department of Medicine, University of Calgary, Calgary, Canada; 3 Department of Medicine, Johns Hopkins School of Medicine, Baltimore, Maryland; 4 Department of Medicine, School of Medicine, University of North Carolina at Chapel Hill; 5 Department of Epidemiology, University of North Carolina at Chapel Hill; 6 Stanford Center for Population Health Sciences, Palo Alto, California; 7 Department of Health Policy and Management, Yale School of Public Health, New Haven, Connecticut; 8 Department of Medicine, Yale School of Medicine, New Haven, Connecticut; 9 VA Connecticut Healthcare System, West Haven; 10 Kaiser Permanente Mid-Atlantic States, Mid-Atlantic Permanente Research Institute, Rockville, Maryland; 11 Division of Research, Kaiser Permanente Northern California, Oakland; 12 Department of Infectious Diseases, Oakland Medical Center, Oakland, California; 13 Epidemiology Branch, Division of AIDS at National Institute of Allergy and Infectious Diseases, National Institutes of Health, Rockville, Maryland

## Abstract

**Importance:**

Recommendations for additional doses of COVID-19 vaccines for people with HIV (PWH) are restricted to those with advanced disease or unsuppressed HIV viral load. Understanding SARS-CoV-2 infection risk after vaccination among PWH is essential for informing vaccination guidelines.

**Objective:**

To estimate the rate and risk of breakthrough infections among fully vaccinated PWH and people without HIV (PWoH) in the United States.

**Design, Setting, and Participants:**

This cohort study used the Corona-Infectious-Virus Epidemiology Team (CIVET)-II (of the North American AIDS Cohort Collaboration on Research and Design [NA-ACCORD], which is part of the International Epidemiology Databases to Evaluate AIDS [IeDEA]), collaboration of 4 prospective, electronic health record–based cohorts from integrated health systems and academic health centers. Adult PWH who were fully vaccinated prior to June 30, 2021, were matched with PWoH on date of full vaccination, age, race and ethnicity, and sex and followed up through December 31, 2021.

**Exposures:**

HIV infection.

**Main Outcomes and Measures:**

COVID-19 breakthrough infections, defined as laboratory evidence of SARS-CoV-2 infection or COVID-19 diagnosis after a patient was fully vaccinated.

**Results:**

Among 113 994 patients (33 029 PWH and 80 965 PWoH), most were 55 years or older (80 017 [70%]) and male (104 967 [92%]); 47 098 (41%) were non-Hispanic Black, and 43 218 (38%) were non-Hispanic White. The rate of breakthrough infections was higher in PWH vs PWoH (55 [95% CI, 52-58] cases per 1000 person-years vs 43 [95% CI, 42-45] cases per 1000 person-years). Cumulative incidence of breakthroughs 9 months after full vaccination was low (3.8% [95% CI, 3.7%-3.9%]), albeit higher in PWH vs PWoH (4.4% vs 3.5%; log-rank *P* < .001; risk difference, 0.9% [95% CI, 0.6%-1.2%]) and within each vaccine type. Breakthrough infection risk was 28% higher in PWH vs PWoH (adjusted hazard ratio, 1.28 [95% CI, 1.19-1.37]). Among PWH, younger age (<45 y vs 45-54 y), history of COVID-19, and not receiving an additional dose (aHR, 0.71 [95% CI, 0.58-0.88]) were associated with increased risk of breakthrough infections. There was no association of breakthrough with HIV viral load suppression, but high CD4 count (ie, ≥500 cells/mm^3^) was associated with fewer breakthroughs among PWH.

**Conclusions and Relevance:**

In this study, COVID-19 vaccination, especially with an additional dose, was effective against infection with SARS-CoV-2 strains circulating through December 31, 2021. PWH had an increased risk of breakthrough infections compared with PWoH. Expansion of recommendations for additional vaccine doses to all PWH should be considered.

## Key Points

**Question:**

Are the rate and risk of COVID-19 breakthrough infections higher among vaccinated people with vs without HIV in the United States through December 31, 2021?

**Findings:**

In this cohort study of 113 994 patients, risk of breakthrough infection was low overall (3.8%) but 28% higher in people with vs without HIV. The breakthrough rate was also higher in people with vs without HIV (55 cases per 1000 person-years vs 43 cases per 1000 person-years).

**Meaning:**

The higher rate and risk of infection in people with HIV observed in this study suggests comprehensive inclusion of this population in recommendations for additional primary doses in immunocompromised groups.

## Teaser

This cohort study estimates the rate and risk of breakthrough COVID-19 infections among fully vaccinated people with and without HIV in the United States.

## Introduction

COVID-19 breakthrough infections are occurring in a small percentage of vaccinated individuals in the United States, observed in both clinical trials^1,2,3^ and observational settings.^4,5,6,7,8,9^ Breakthrough infections can help us understand the utility of vaccines against SARS-CoV-2. Evidence on breakthrough risk can inform public health policies, including recommendations of additional primary doses. Prior studies have not been consistent in indicating an increased risk of breakthrough infection in people with HIV (PWH), an immunocompromised population, vs people without HIV (PWoH); these studies were limited in geographic scope, number of PWH, and did not consider HIV viremia or CD4 T-lymphocyte count.^10,11,12^ Vaccine clinical trials were insufficiently powered to stratify outcomes by HIV status.^1,2,3^

The US Centers for Disease Control and Prevention (CDC) recommends booster vaccines for adults. For PWH, recommendations for additional primary doses 28 days after a second dose of the mRNA-1273 (Moderna) or BNT162 (Pfizer) vaccines or first dose of Ad26.COV2.S (Janssen) applies only to those with advanced or untreated HIV.^13^ People with treated HIV may have chronic immune impairment or persistent immune activation without advanced HIV, which could increase risk for breakthrough infections.^14^ Generalizable evaluations of breakthrough infections are needed to inform US vaccine guidelines on the primary vaccine schedule for all PWH. We sought to determine whether HIV status was associated with increased rate or risk of COVID-19 breakthrough infection among fully vaccinated patients in the United States by vaccination type and, among PWH, by immune and viral suppression status.

## Methods

### Study Population

The Corona-Infectious-Virus Epidemiology Team (CIVET)-II cohort comprises 4 cohorts from integrated health systems and academic health centers contributing longitudinal data to the North American AIDS Cohort Collaboration on Research and Design.^15^ CIVET-II is part of the North American AIDS Cohort Collaboration on Research and Design (NA-ACCORD) of the International Epidemiology Databases to Evaluate AIDS (IeDEA). The contributing centers include Kaiser Permanente Mid-Atlantic States (Maryland, District of Columbia, northern Virginia); Kaiser Permanente Northern California; University of North Carolina Chapel Hill HIV Clinic; and the Veterans Aging Cohort Study (VACS), a sample of all PWH receiving care within the National US Veterans Affairs Healthcare System. Cohorts received approval (including waivers and/or exemptions of consent when necessary) from their local institutional review boards (IRB). Overall approval was obtained from the Johns Hopkins Bloomberg School of Public Health IRB.

Patients included adult (≥18 years old) PWH and PWoH, who were in-care (defined in eTable 1 in the Supplement) and fully vaccinated against COVID-19 with a vaccine authorized in the United States (first date of which was December 11, 2020) by June 30, 2021. Full vaccination status was defined as: (1) 14 days after the second dose of BNT162 or mRNA-1273 vaccines or (2) 14 days after the single dose of the Ad26.COV2.S viral vector vaccine.^16^ Patients were excluded if their primary series mRNA vaccine types were discrepant.

Each fully vaccinated PWH was matched (without replacement) to 3 fully vaccinated PWoH on the date fully vaccinated (closest date within 14 days), 10-year age group (18-24, 25-34, 35-44, 45-54, 55-64, 65-74, or ≥75 years), race and ethnicity (Asian, Black or African American, Hispanic, other, unknown, or White), and sex at birth (female or male). Race and ethnicity were included given the disproportionate burden of HIV and potential disproportionate burden of breakthrough COVID-19 infections by race and ethnicity. PWH could be matched to patients either 1 age group above or below their category or matched to 2 PWoH when 3 were not available. All cohorts completed this matching scheme except for VACS (67 627 participants), which matched each veteran with HIV to 2 veterans without HIV on age, race and ethnicity, sex, and clinical site at enrollment into VACS; VACS patients were not matched on vaccination date.^17^

All variables were abstracted from electronic health records. Our study follows the Strengthening the Reporting of Observational Studies in Epidemiology (STROBE) reporting guidelines.

### Outcome: Breakthrough Infection

The first SARS-CoV-2 infection or COVID-19 illness diagnosed after the date an individual was fully vaccinated was defined as a breakthrough case (eFigure 1 in the Supplement). Incident COVID-19 cases were identified using (1) positive or detectable SARS-CoV-2 nucleic acid amplification assay or antigen test and/or (2) *International Statistical Classification of Diseases and Related Health Problems, 10th Revision* (*ICD-10*) diagnosis codes (eFigure 1 in the Supplement).

### Exposure: HIV Infection

PWH were identified using HIV registries or *ICD-10* diagnosis codes for HIV, depending on the cohort (eTable 1 in the Supplement). PWoH were classified as such if there was no evidence of HIV infection using these sources as of December 11, 2020.

### Covariates

In addition to demographic factors, covariates included the type of primary series vaccine (BNT162, mRNA-1273, or Ad26.COV2.S ), additional vaccine dose after full vaccination, and evidence of SARS-CoV-2 infection prior to the date fully vaccinated (history of COVID-19). Additional vaccine dose was defined as any vaccine dose 28 days or more after the second primary mRNA dose or Ad26.COV2.S single dose. COVID-19 diagnoses prior to full vaccination included any infection prior to the date fully vaccinated.

Among PWH, CD4 count and suppressed HIV-1 RNA (<50 copies/mL, the highest lower limit of detection across cohorts) were collected as close to full vaccination date as possible (within a window of January 1, 2020, to full vaccination) and at antiretroviral therapy (ART) initiation (within a window of 12 months prior to 1 month after). History of AIDS was defined as clinical diagnosis^18^ and/or CD4 count less than 200 cells/mm^3^ prior to date fully vaccinated.

### Statistical Analysis

Study entry for eligible patients was the date fully vaccinated. Patients were followed up until date of breakthrough infection, death, disenrollment (applicable to the 2 Kaiser Permanente health systems), 275 days (9 months) after fully vaccinated date, or December 31, 2021, whichever occurred first. Given patients could not meet the definition of fully vaccinated until January 18 (for BNT162 recipients), January 22 (for mRNA-1273 recipients), or March 13 (for Ad26.COV2.S recipients (eTable 2 in the Supplement), follow-up ended 9 months after full vaccination to avoid unstable risk sets.

We assessed the distribution of demographic and clinical characteristics to determine differences between PWH and PWoH. Unable to discriminate between additional primary vs booster doses, we assessed differences among PWH by timing of third dose using a threshold of less than 5 months vs 5 months or more, reflective of current booster guidelines (eTable 3 in the Supplement).

Incidence rates and 95% CIs of COVID-19 breakthrough infections after the date fully vaccinated were calculated per 1000 person-years for each month overall, by HIV status, and by vaccine type. The 9-month cumulative incidence of breakthrough infections by HIV status was estimated from the date fully vaccinated and stratified by HIV status and, among PWH, by CD4 count (<200, 200-349, 350-499, and ≥500 cells/mm^3^) and HIV viral suppression status. Cumulative incidence was estimated by HIV status for each vaccine type. Log-rank tests were calculated to test for differences in cumulative incidence.

We compared the risk of breakthrough infection by HIV status using Cox proportional hazard models (estimating the cause-specific hazard) to assess unadjusted hazard ratios (HRs) and adjusted hazard ratios (aHRs) with 95% CIs. Adjustment factors included sex, race and ethnicity, age, primary vaccine series type, third vaccine dose (time-varying), cohort, and an interaction between history of COVID-19 and 3-month calendar period (January-March, April-June, July-September, and October-December 2021). We adjusted for cohort as a surrogate for sociodemographic and regional differences in COVID-19 transmission rates, vaccine rollout, COVID-19 testing protocols, and access to care. An interaction term with calendar period was included due to nonproportional hazards by history of COVID-19 and improved model fit.

Among PWH, CD4 count and suppressed viral load were assessed as risk factors for breakthrough infection while accounting for the covariates included in the main analysis of PWH and PWoH. Subgroup analyses included (1) excluding those with a history of COVID-19 prior to full vaccination and (2) excluding VACS patients because of the differences in matching strategies.

Analyses were conducted with R version 4.1.2 (R Project for Statistical Computing). A 2-sided *P* < .05 guided statistical significance interpretation.

## Results

### Study Population Characteristics

After excluding 131 patients (52 PWH and 79 PWoH) with discrepant primary series mRNA vaccine types, 113 994 patients (33 029 PWH and 80 965 PWoH) were observed, most of whom were 55 years or older (80 017 [70%]) and male (104 967 [92%]), with 47 098 (41%) non-Hispanic Black and 43 218 (38%) non-Hispanic White individuals (Table 1). Most received BNT162 (58 360 [51%]) or mRNA-1273 (48 145 [42%]) vaccines; 7489 (7%) received Ad26.COV2.S. PWH were more likely to receive an additional dose than PWoH (18 385 [56%] vs 36 267 [45%]). Receiving an additional dose of a vaccine that was different than the primary series was most common among those receiving Ad26.COV2.S (1676 [67%]), followed by mRNA-1273 (1040 [5%]), and BNT162 (1154 [4%]). Additional doses predominantly occurred 5 months or more after full vaccination (eFigure 2 in the Supplement). Differences in PWH who did and did not receive an additional vaccine dose are detailed in eTable 3 in the Supplement. Among PWH, 8335 (25%) had a history of AIDS prior to vaccination. At full vaccination, 26 052 (91%) had viral suppression and had a median (IQR) CD4 count of 636 (449-858) cells/mm^3^ (Table 1).

**Table 1.** Characteristics at Date Fully Vaccinated of 113 994 PWH and PWoH

Characteristic	Patients, No. (%)^a^		
	Overall (N = 113 994)	PWoH (n = 80 965)	PWH (n = 33 029)
Age, y			
18-24	318 (0.3)	230 (0.3)	88 (0.3)
25-34	4577 (4.0)	2963 (3.7)	1614 (4.9)
35-44	10 521 (9.2)	7096 (8.8)	3425 (10.4)
45-54	18 561 (16.3)	12 795 (15.8)	5766 (17.5)
55-64	37 018 (32.5)	26 020 (32.1)	10 998 (33.3)
65-74	32 902 (28.9)	24 327 (30.0)	8575 (26.0)
≥75	10 097 (8.9)	7534 (9.3)	2563 (7.8)
Sex			
Male	104 967 (92.1)	74 291 (91.8)	30 676 (92.9)
Female	9027 (7.9)	6674 (8.2)	2353 (7.1)
Race and ethnicity			
Hispanic	15 084 (13.2)	10 980 (13.6)	4104 (12.4)
Non-Hispanic			
Asian	3906 (3.4)	2872 (3.5)	1034 (3.1)
Black or African American	47 098 (41.3)	33 553 (41.4)	13 545 (41.0)
White	43 218 (37.9)	30 250 (37.4)	12 968 (39.3)
Other	3562 (3.1)	2486 (3.1)	1076 (3.3)
Unknown	1126 (1.0)	824 (1.0)	302 (0.9)
Month fully vaccinated, 2021			
January	1226 (1.1)	867 (1.1)	359 (1.1)
February	12 110 (10.6)	8309 (10.3)	3801 (11.5)
March	29 666 (26.0)	20 964 (25.9)	8702 (26.3)
April	43 105 (37.8)	30 799 (38.0)	12 306 (37.3)
May	20 969 (18.4)	15 176 (18.7)	5793 (17.5)
June	6918 (6.1)	4850 (6.0)	2068 (6.3)
Vaccination series type^b^			
BNT162 (2 doses)	28 806 (25.3)	21 510 (26.6)	7296 (22.1)
BNT162 with third dose			
<5 mo after second dose	1201 (1.1)	316 (0.4)	885 (2.7)
≥5 mo after second dose	28 353 (24.9)	19 145 (23.6)	9208 (27.9)
mRNA-1273 (2 doses)	25 563 (22.4)	19 483 (24.1)	6080 (18.4)
mRNA-1273 with third dose			
<5 mo after second dose	902 (0.8)	242 (0.3)	660 (2.0)
≥5 mo after second dose	21 680 (19.0)	14 791 (18.3)	6889 (20.9)
Ad26.COV2.S (1 dose)	4973 (4.4)	3705 (4.6)	1268 (3.8)
Ad26.COV2.S with second dose			
<5 mo after first dose	92 (0.1)	55 (0.1)	37 (0.1)
≥5 mo after first dose	2424 (2.1)	1718 (2.1)	706 (2.1)
Additional dose			
<5 mo after primary series	2195 (1.9)	613 (0.8)	1582 (4.8)
≥5 mo after primary series	52 457 (46.0)	35 654 (44.0)	16 803 (50.9)
COVID-19 prior to fully vaccinated	6865 (6.0)	4593 (5.7)	2272 (6.9)
CD4 at ART initiation, cells/mm^3^	NA	NA	368.00 (202.00-584.00)
Unknown	NA	NA	14 910 (45.1)
AIDS before fully vaccinated	NA	NA	8335 (25.2)
Unknown (not defined)	NA	NA	0
CD4 at fully vaccinated, cells/mm^3^	NA	NA	636.00 (449.00-858.00)
Unknown	NA	NA	6393 (19.4)
Viral suppression (<50 copies/mL) HIV RNA at fully vaccinated	NA	NA	26 052 (90.5)
Unknown	NA	NA	4240 (12.8)

Abbreviations: ART, antiretroviral therapy; NA, not applicable; PWH, people with HIV; PWoH, people without HIV.

^a^ *P* values for all demographic characteristics were statistically significantly different comparing PWH vs PWoH.

^b^ Vaccine series type denotes the primary vaccination series received; additional dose type does not necessarily match the primary series type.

### Incidence Rates and Risk of Breakthrough Infections

**Figure 1.**
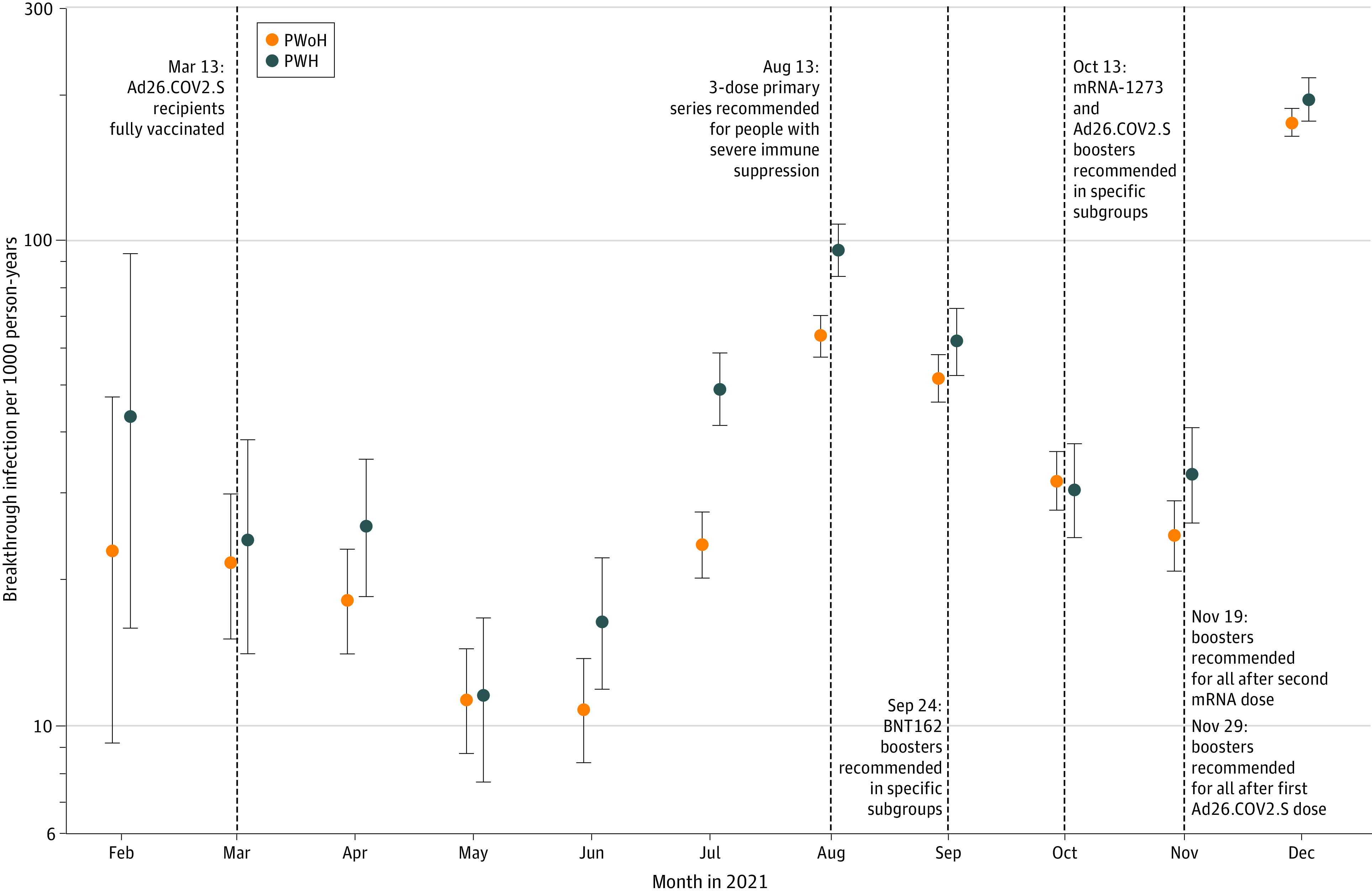
Trends in SARS-CoV-2 Vaccine Breakthrough Incidence Rates Among People With HIV (PWH) and People Without HIV (PWoH) Overall, 113 994 individuals were included. The incidence rate estimates for January 2021 were 0 (95% CI, 0.0-492.3) cases per 1000 person-years for PWH and 57.0 (1.4-317.8) cases per 1000 person-years in PWoH; these estimates are not included in the plot due to small numbers.

The incidence rate of breakthrough infections was 47 (95% CI, 45-48) cases per 1000 person-years (3649 breakthroughs among 78 280 person-years) and was higher in PWH (55 [95% CI, 52-58] cases per 1000 person-years) vs PWoH (43 [95% CI, 42-45] cases per 1000 person-years). The distribution of breakthroughs was congruent with waves of COVID-19 infection in the US in 2021 (Figure 1). The breakthrough rate was highest with the Ad26.COV2.S vaccine (70 [95% CI, 63-78] cases per 1000 person-years), followed by BNT162 (54 [95% CI, 52-56] cases per 1000 person-years), and mRNA-1273 (34 [95% CI, 32-36] cases per 1000 person-years) (eTable 4 in the Supplement). Stratified by vaccine type, the rate of breakthroughs was consistently higher among PWH vs PWoH (eTable 4 in the Supplement).

**Figure 2.**
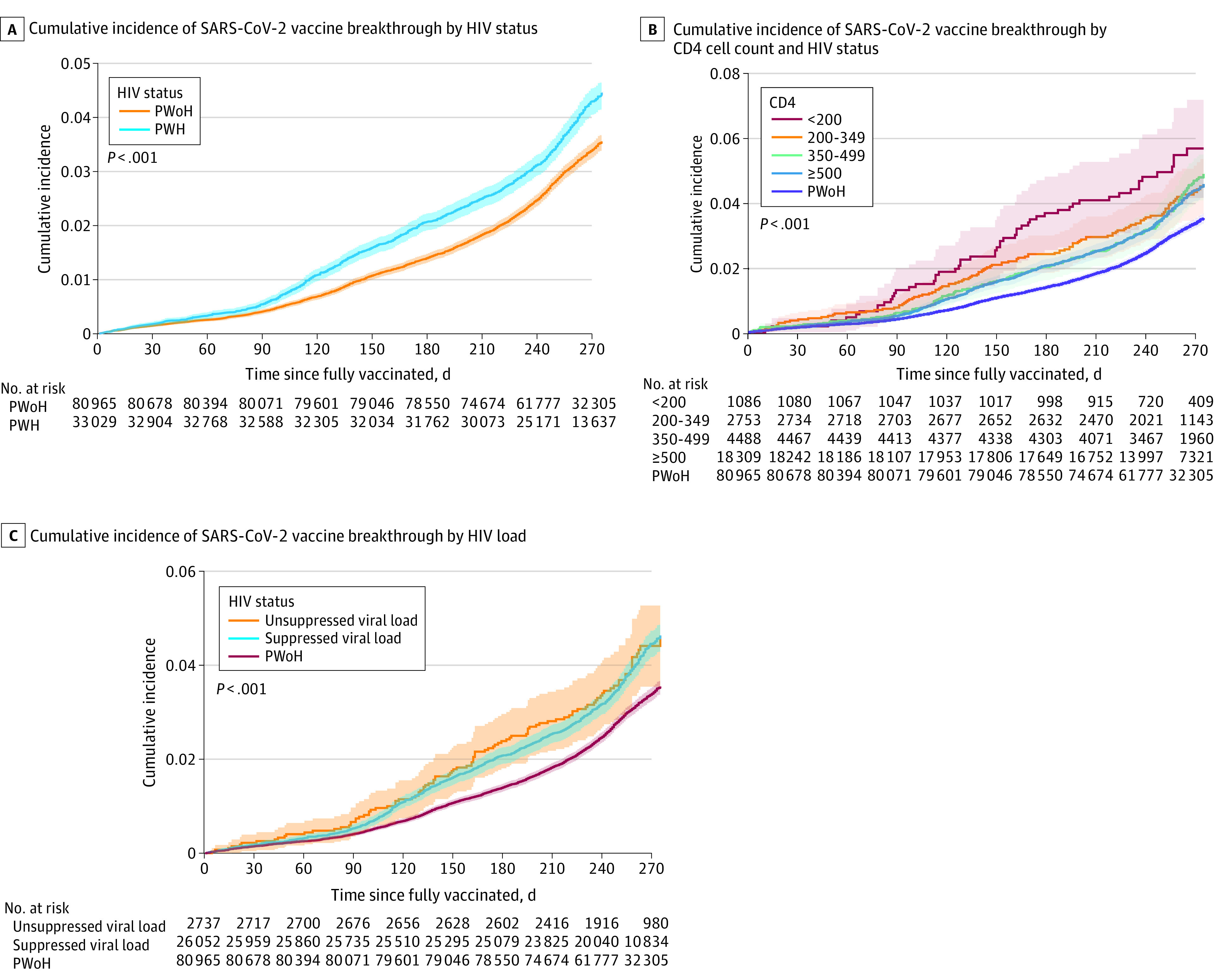
Cumulative Incidence of SARS-CoV-2 Vaccine Breakthrough Stratified by HIV Status, CD4 Count and HIV Status, and HIV Viral Suppression and HIV Status B, Log-rank *P* = .18 after excluding people without HIV (PWoH). C, Log-rank *P* = .47 after excluding PWoH. Viral suppression defined by undetectable HIV-1 RNA viral load (<50 copies/mL). Shaded areas indicate 95% CIs. PWH indicates people with HIV.

The cumulative incidence of breakthrough infection at 275 days (9 months) after full vaccination was 3.8% (95% CI, 3.7%-3.9%) (Figure 2A), and higher among PWH vs PWoH (4.4% [95% CI, 4.2%-4.7%] vs 3.5% [95% CI, 3.4%-3.7%]; log-rank *P* < .001), yielding a risk difference of 0.9% (95% CI, 0.6%-1.2%). PWH with lower CD4 counts at full vaccination had higher cumulative incidence of breakthroughs, although this was not statistically significant (log-rank *P* = .17 after excluding PWoH) (Figure 2B). Breakthrough risk was not significantly higher in PWH with unsuppressed vs suppressed HIV (log-rank *P* = .80 after excluding PWoH) (Figure 2C). PWH had higher cumulative incidence of breakthrough regardless of CD4 count or HIV viral load suppression compared to PWoH (Figure 2B and C).

**Figure 3.**
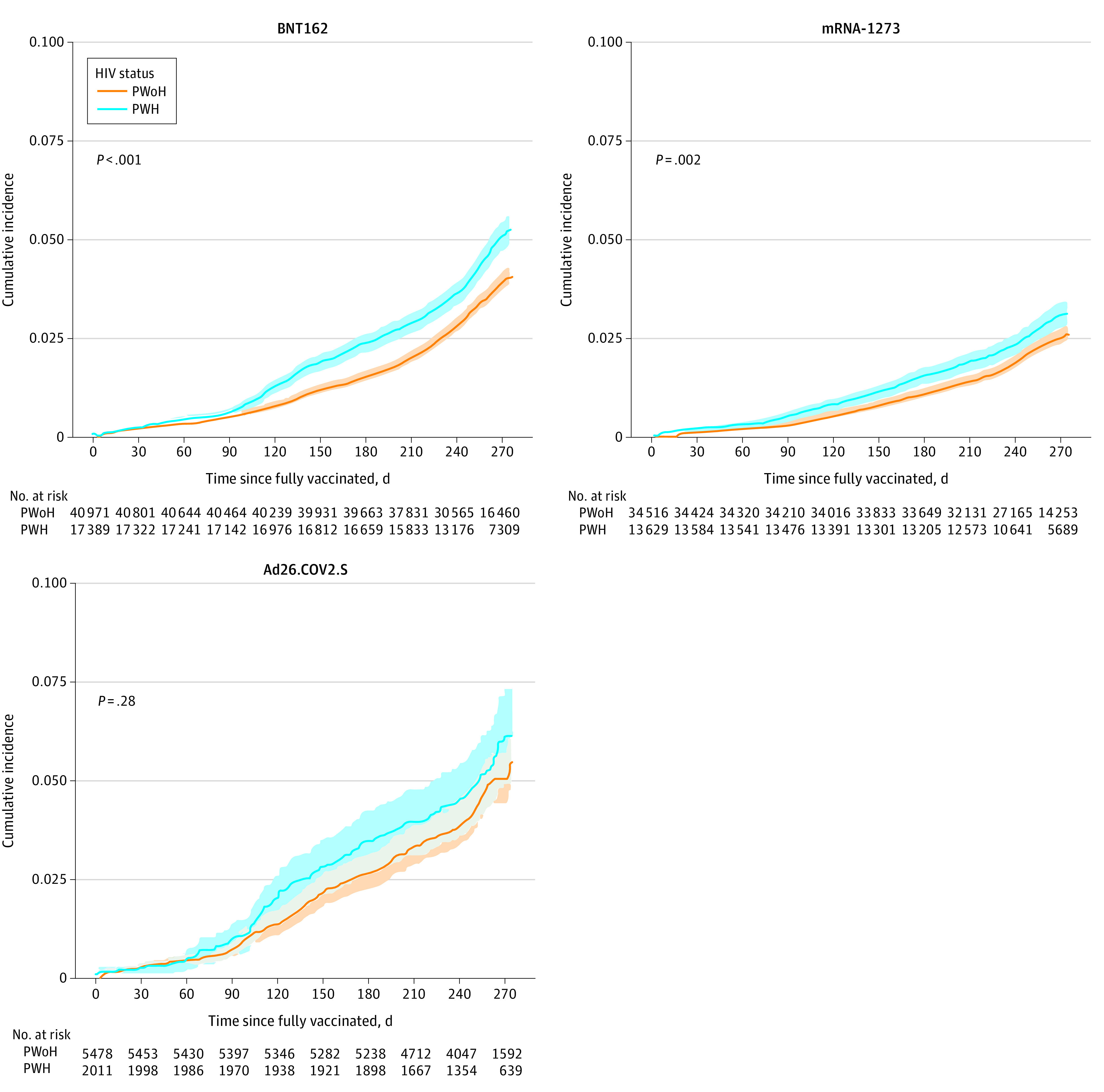
Cumulative Incidence of SARS-CoV-2 Vaccine Breakthrough Infection Stratified by HIV Status and Primary Vaccination Series Type Shaded areas indicate 95% CIs; PWH, people with HIV; PWoH, people without HIV.

The overall risk of breakthrough was highest with Ad26.COV2.S (5.7% [95% CI, 5.1%-6.3%]), followed by BNT162 (4.4% [95% CI, 4.2%-4.6%]) and mRNA-1273 (2.8% [95% CI, 2.6%-2.9%]). Within each primary series vaccine type, the risk was higher among PWH vs PWoH (Figure 3).

### Factors Associated With Risk of Breakthrough

PWH had a significantly higher risk of breakthrough infection compared with PWoH (aHR, 1.28 [95% CI, 1.19-1.37]) after adjusting for covariates (Table 2). The association was robust in subgroup analyses in which (1) patients with of history of COVID-19 were excluded (107 129 patients after exclusion; aHR, 1.30 [95% CI, 1.20-1.40]) and (2) VACS patients were excluded (46 367 patients after exclusion; aHR, 1.34 [95% CI, 1.21-1.48]).

**Table 2.** Crude and Adjusted HRs for SARS-CoV-2 Vaccination Breakthrough Infections

Characteristic	HR (95% CI)	
	Crude	Adjusted
**Among PWH and PWoH (n = 113 994)** ^a^		
HIV status		
PWoH	1 [Reference]	1 [Reference]
PWH	1.27 (1.18-1.36)	1.28 (1.19-1.37)
**Among PWH (n = 33 029)** ^b^		
Sex		
Male	1 [Reference]	1 [Reference]
Female	1.21 (0.97-1.50)	0.94 (0.75-1.19)
Race and ethnicity		
Hispanic	1.46 (1.21-1.76)	1.13 (0.93-1.37)
Non-Hispanic		
Asian	1.17 (0.81-1.70)	0.85 (0.58-1.23)
Black or African American	1.35 (1.18-1.55)	1.14 (0.98-1.32)
White	1 [Reference]	1 [Reference]
Other	0.91 (0.61-1.37)	0.84 (0.56-1.26)
Unknown	1.06 (0.52-2.13)	0.75 (0.37-1.52)
Age, y		
18-24	2.99 (1.53-5.82)	2.64 (1.35-5.17)
25-34	1.80 (1.42-2.29)	1.70 (1.33-2.18)
35-44	1.35 (1.10-1.66)	1.31 (1.06-1.61)
45-54	1 [Reference]	1 [Reference]
55-64	0.74 (0.62-0.89)	0.78 (0.65-0.93)
65-74	0.65 (0.54-0.79)	0.70 (0.57-0.85)
≥75	0.54 (0.40-0.72)	0.57 (0.42-0.78)
Vaccination series type		
mRNA-1273, 2 doses	0.59 (0.51-0.69)	0.66 (0.57-0.77)
mRNA-1273 with third dose	0.43 (0.32-0.57)	0.50 (0.38-0.67)
BNT162, 2 doses	1 [Reference]	1 [Reference]
BNT162 with third dose	0.67 (0.54-0.82)	0.71 (0.58-0.88)
Ad26.COV2.S	1.18 (0.92-1.50)	1.14 (0.89-1.46)
Ad26.COV2.S with second dose	0.61 (0.29-1.30)	0.60 (0.28-1.27)
COVID-19 prior to full vaccination		
No	1 [Reference]	1 [Reference]
Yes	2.29 (1.93-2.72)	1.96 (1.65-2.33)
Calendar period		
January-March	1.45 (0.85-2.48)	1.68 (0.99-2.85)
April-June	1 [Reference]	1 [Reference]
July-September	3.15 (2.29-4.33)	2.59 (1.89-3.55)
October-December	1.72 (1.17-2.53)	1.26 (0.86-1.85)
HIV RNA, copies/mL		
Unsuppressed, ≥50	1 [Reference]	1 [Reference]
Suppressed, <50	0.97 (0.79-1.19)	1.03 (0.84-1.28)
CD4 count at fully vaccinated, cells/mm^3^		
<200	1 [Reference]	1 [Reference]
200-349	0.75 (0.54-1.04)	0.78 (0.56-1.08)
350-499	0.75 (0.55-1.02)	0.75 (0.55-1.02)
≥500	0.72 (0.55-0.95)	0.66 (0.50-0.88)

Abbreviations: HR, hazard ratio; PWH, people with HIV; PWoH, people without HIV.

^a^ Adjusted for age, sex, race and ethnicity, primary vaccination series type, COVID-19 prior to full vaccination, 3-month calendar period, an interaction of COVID-19 prior to fully vaccinated and 3-month calendar period, and cohort.

^b^ Overall, 10 633 PWH (32% of all) were excluded because of missing CD4 or HIV RNA measurements. Adjusted for the covariates in the table and cohort.

Among PWH, older age (≥55 years) was associated with decreased risk of breakthrough, and younger age (<44 years) was associated with increased risk, compared with patients ages 44 to 54 (Table 2). Compared with patients receiving BNT162, patients who received the mRNA-1273 primary series had a reduced risk of breakthrough infection (aHR, 0.66 [95% CI, 0.57-0.77]), which strengthened for those with mRNA-1273 plus an additional dose of any type (aHR, 0.50 [95% CI, 0.38-0.67]). An additional dose following BNT162 primary vaccination was associated with lower risk than the 2 doses of BNT162 (aHR, 0.71 [95% CI, 0.58-0.88]). The risk of breakthrough infection was higher during the Delta variant (B.1.617.2) surge from July to September 2021 relative to April to June 2021 (Table 2).

There was no association between breakthrough risk and HIV viral suppression among PWH. CD4 count of 500 or greater (vs <200) cells/mm^3^ was associated with decreased breakthrough risk (aHR, 0.66 [95% CI, 0.50-0.88]). There was a 2-fold increase in the risk of breakthrough among those with evidence of a history of COVID-19 (aHR, 1.96 [95% CI, 1.65-2.33]). Excluding 24 484 patients with a history of COVID-19, the associations of age, vaccine type, 3-month calendar period, unsuppressed viral load, and CD4 count were similar (data not shown). After removing 9517 VACS patients, there was no association between history of COVID-19 and breakthrough risk (aHR, 0.80 [95% CI, 0.56-1.13]), and the associations of age, calendar period, and viral suppression were comparable with the main model; Ad26.COV2.S plus an additional dose was associated with reduced risk of breakthrough (vs 2 doses of BNT162: aHR, 0.19 [95% CI, 0.05-0.78]), and the association with a CD4 count of 500 or greater cells/mm^3^ (vs <200 cells/mm^3^) was null (aHR, 0.94 [95% CI, 0.53-1.65]).

## Discussion

Among 113 994 fully vaccinated patients receiving care at 4 academic or integrated health care systems across varied geographic regions in the United States, breakthroughs were uncommon in PWH and PWoH 9 months after full vaccination (3.8%), demonstrating vaccine effectiveness against SARS-CoV-2 variants circulating prior to December 31, 2021. There was a consistently higher rate of breakthrough infections among PWH (compared with PWoH), suggesting a higher risk of breakthroughs in PWH after adjustment for demographic and clinical factors and vaccine type. Breakthrough cumulative incidence was higher in PWH (vs PWoH) irrespective of CD4 count of viral suppression. Receipt of any vaccine dose after primary series conferred further protection against breakthrough infection among PWH, exhibiting the importance of boosters and additional primary doses.

Two prior observational studies found no association between HIV status and breakthrough infection risk.^10,11^ In our analysis, with a large study population across several US geographic regions followed longitudinally to enable a time-to-event analysis, we found a 28% increased risk of breakthrough infection in PWH vs PWoH. Discrepancies in findings are likely because of greater precision in our study and varying calendar periods of observation, reflecting SARS-CoV-2 variant differences in transmissibility. A more recent analysis with larger sample size, a study period from December 10, 2020, to September 16, 2021 (prior to the uptake of boosters and emergence of Omicron), wide US geographic distribution, and also reliant on electronic health record data, reported an adjusted incidence rate ratio of breakthroughs in PWH (vs PWoH) of 1.33 (95% CI, 1.18-1.49), suggesting an increased risk of breakthrough in PWH.^12^ Our findings are consistent with studies that have found an increased risk of breakthroughs among those with immunocompromising conditions.^10,12,19,20,21,22^

Our observation of differential breakthrough risk by vaccine type is consistent with studies showing lower effectiveness for Ad26.COV2.S relative to mRNA vaccines,^23^ and among mRNA vaccines, more breakthroughs among those with BNT162 vs mRNA-1273 primary series.^10,11,24^ We also saw a steep increase in the rate of breakthrough infections in December in both PWH and PWoH owing to circulation of the Omicron variant. This should be considered an underestimate of the December breakthrough rate due to testing limitations in many parts of the United States and potentially greater use of at-home testing, which may not be reported.

Among PWH, we suspect the inverse association between older age and breakthrough risk may be because of behavioral modifications by older patients, including adoption of masking and social distancing.^25,26^ The association we observed between history of COVID-19 and increased breakthrough risk may reflect increased exposure and/or varying adoption of prevention measures. PWH with increased exposure prior to vaccination may have persistent increased exposure after full vaccination, leading to increased breakthroughs. Alternatively, this may reflect the increased burden of underlying comorbidities among people aging with HIV. Detecting COVID-19 prior to, and after, being fully vaccinated may also be a function of lower barriers to accessing and regularly seeking care. Finally, breakthrough risk varied by calendar period, aligning with the surge of infections and Omicron variant.^27^ Through these variations over time, the cumulative incidence of breakthroughs was higher among PWH vs PWoH regardless of CD4 count, suggesting residual immune function abnormalities (despite CD4 count recovery) results in an increased risk of breakthroughs for all PWH, not only those with advanced HIV disease or unsuppressed HIV RNA.

### Limitations

This study has limitations. Our findings are not generalizable to all PWH in the United States. Our study population had a greater proportion of men (92%) than found in the US population of PWH, and those without regular access to health care (who may be at greater risk for COVID-19 infection) were less likely to be included. Individuals engaged in care may have greater health-seeking behaviors, including greater frequency of COVID-19 testing, leading to higher detection of breakthrough infections than in the general population. Differentially higher detection of breakthroughs in PWH vs PWoH may exist because of (1) increased severity of breakthrough in PWH vs PWoH,^28,29^ as symptomatic disease is identified more frequently than asymptomatic disease, or (2) PWH may have detectable SARS-CoV-2 virus 90 days or more after infection, as demonstrated in a case report of a PWH with advanced disease and others who are immunocompromised.^30,31,32^ Future analyses should account for testing practices and include a larger proportion of women with HIV. Although our matching scheme was not consistent across cohorts, distributions of matching factors indicate that our samples of PWH and PWoH were comparable. We included matching factors in multivariable analyses to address residual confounding and account for the matching approaches. We were unable to account for comorbidities, which are common in aging PWH and could affect breakthrough risk, likelihood of receiving an additional dose, and COVID-19 testing frequency. We do not describe the severity of COVID-19 breakthrough illness in PWH vs PWoH. Our findings may not be generalizable to future SARS-CoV-2 variants, but our study includes calendar time when Alpha, Delta, and Omicron variants were circulating.

## Conclusions

For PWH with advanced or untreated HIV, the CDC currently recommends an additional dose 28 days or more after the second mRNA dose or the first Ad26.COV2.S dose, and a booster 3 months or more for mRNA or 2 months or more for those initially vaccinated with Ad26.COV2.S dose.^13^ Our findings indicate all PWH may benefit from being included in this recommendation, as the risk of breakthrough was higher in PWH than PWoH regardless of CD4 count (reflecting advanced disease) or HIV viral suppression (reflecting treatment). A first booster dose is now recommended for those aged 12 years and older, and a second booster is recommended for those aged 50 years and older and certain immunocompromised groups; our findings suggest these booster doses may be even more important for all PWH to prevent breakthrough infections, although further research is needed to study this potential association. Ultimately, policy makers must determine the appropriate balance between preventing further COVID-19 infections and possibly unnecessary additional vaccinations. Increased risk of breakthrough infections in PWH merits continued monitoring as the pandemic persists, immunity to primary vaccine series wanes, boosters are widely recommended, and new variants emerge.

## Notes

Supplement. **eTable 1.** Definitions of in Care and Criteria Used to Identify People With and Without HIV **eFigure 1. **Schematic for Identification of SARS-CoV-2 Breakthrough Infections **eTable 2.** Timeline of Vaccination Availability and Vaccine Guidelines Recommendations, December 2020 to December 2021 **eFigure 2. **Distribution of Time From Fully Vaccinated to Receipt of Another Dose of Vaccine, by HIV Status, Among 133 994 Patients **eTable 3.** Characteristics of 33 029 PWH Who Did and Did Not Receive a Vaccine Dose After Their Primary Series **eTable 4. **Breakthrough Infection Incidence Rates and 95% CIs, by Vaccine Type and HIV Status
