# Supplementary material for: Analysis of Postvaccination Breakthrough COVID-19 Infections Among Adults With HIV in the United States
Source: JAMA Netw Open. 2022 Jun 7;5(6):e2215934. doi: 10.1001/jamanetworkopen.2022.15934 (PMC9175076; doi:10.1001/jamanetworkopen.2022.15934)
Supplement: Supplement. — eTable 1. Definitions of in Care and Criteria Used to Identify People With and Without HIV eFigure 1. Schematic for Identification of SARS-CoV-2 Breakthrough Infections eTable 2. Timeline of Vaccination Availability and Vaccine Guidelines Recommendations, December 2020 to December 2021 eFigure 2. Distribution of Time From Fully Vaccinated to Receipt of Another Dose of Vaccine, by HIV Status, Among 133 994 Patients eTable 3. Characteristics of 33 029 PWH Who Did and Did Not Receive a Vaccine Dose After Their Primary Series eTable 4. Breakthrough Infection Incidence Rates and 95% CIs, by Vaccine Type and HIV Status [file jamanetwopen-e2215934-s001.pdf]

Supplemental Online Content

Coburn SB, Humes E, Lang R, et al; Corona-Infectious-Virus Epidemiology Team (CIVETs) of the NA-ACCORD of IeDEA. Analysis of postvaccination breakthrough COVID-19 infections among adults with HIV in the United States. *JAMA Netw Open.* 2022;5(6):e2215934. doi:10.1001/jamanetworkopen.2022.15934

eTable 1. Definitions of in Care and Criteria Used to Identify People With and Without HIV

eFigure 1. Schematic for Identification of SARS-CoV-2 Breakthrough Infections

eTable 2. Timeline of Vaccination Availability and Vaccine Guidelines Recommendations, December 2020 to December 2021

eFigure 2. Distribution of Time From Fully Vaccinated to Receipt of Another Dose of Vaccine, by HIV Status, Among 133 994 Patients

eTable 3. Characteristics of 33 029 PWH Who Did and Did Not Receive a Vaccine Dose After Their Primary Series

eTable 4. Breakthrough Infection Incidence Rates and 95% CIs, by Vaccine Type and HIV Status

This supplemental material has been provided by the authors to give readers additional information about their work.

© 2022 Coburn SB et al. *JAMA Network Open.*

eTable 1. Definitions of in Care and Criteria Used to Identify People With and Without HIV

**Cohort**	**Description**	**“In care” or “in cohort”**	**PWH**
Kaiser Permanente Mid-Atlantic States (KPMAS)	Integrated Health system	In Care – KPMAS membership for ≥1 month in 2020	HIV registry*
Kaiser Permanente Northern California (KPNC)	Integrated Health system	In Care – KPNC membership for ≥1 month between 3/1/20 and 12/31/20	HIV registry*
University of North Carolina Chapel Hill (UNC)	Medical center cohort	≥1 encounter with UNC Health in 2019 and alive as of 03/01/2020	HIV diagnosis (by ICD diagnosis code)
Veterans Aging Cohort Study (VACS)	National cohort sampling from all PWH and 1:2 demographically-matched PWoH in care in the Veterans Health Administration (VA) system	Enrolled in VACS from 1996-2017 and alive in 2020	HIV diagnosis (presence of 1 inpatient or 2 outpatient ICD codes for HIV)

*The Kaiser Permanente Mid-Atlantic States and Northern California HIV Registries are databases of members diagnosed with HIV since 1998 and 1980, respectively. Primary sources used to identify HIV patients are HIV-specific laboratory tests, antiretroviral therapy, hospital-based HIV diagnosis, and diagnosis confirmed by infectious disease physicians.

eFigure 1. Schematic for Identification of SARS-CoV-2 Breakthrough Infections

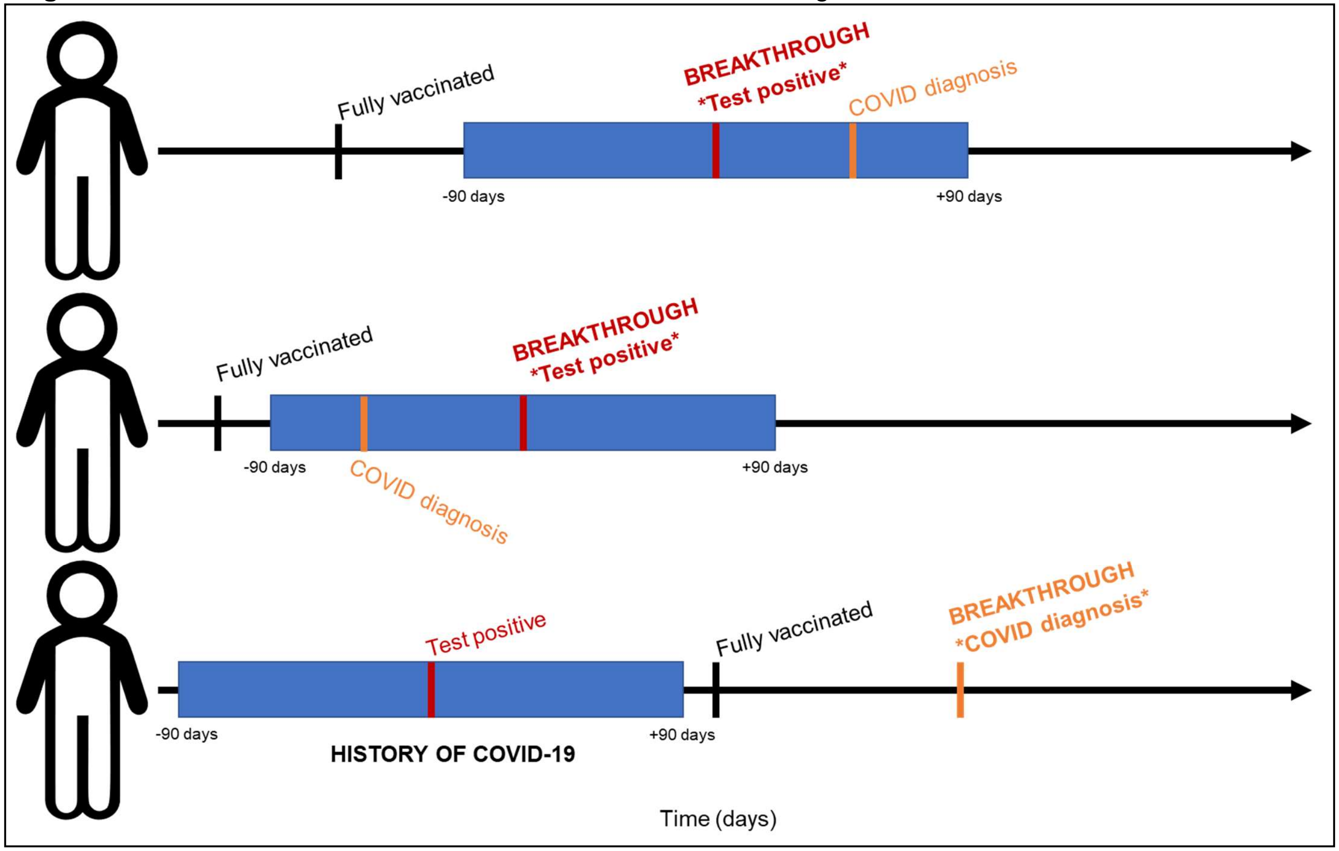

This figure depicts three mock scenarios to demonstrate how we defined breakthrough infection using laboratory data and diagnosis codes. Each "person" demonstrates possible scenarios of when and how laboratory tests were confirmed/diagnoses were made, and how we reconciled these records. For each patient, all positive SARS-CoV-2 laboratory tests were identified. Additional positive laboratory tests and/or diagnoses occurring within +/- 90 days of the date of a positive/detectable SARS-CoV-2 test result were considered persistent infection per CDC suggested classification. In instances where there was a COVID-19 diagnosis within the 90-day window of a positive or detectable test result, the date of the laboratory test was used to define the date of the breakthrough case; positive laboratory results were prioritized over diagnosis codes for calculation of breakthrough date due to their greater specificity. This reconciliation was repeated for diagnoses that did not occur within +/- 90 window of another laboratory test.

Footnotes:

Blue bar indicates +/- 90 window around a positive or detectable SARS-CoV-2 test result.

Red bar/text indicates the date of a positive or detectable SARS-CoV-2 test result.

Orange bar/text indicates the date of a COVID-19 diagnosis (using ICD-10 codes).

Diagnosis codes include: U07.1 (specific to COVID-19), B34.2 (Coronavirus infection, unspecified), B97.21 (SARS-associated coronavirus causing disease classified elsewhere), B97.29 (other coronavirus as the cause of diseases classified elsewhere, or J12.81 (pneumonia due to SARS-associated coronavirus).

© 2022 Coburn SB et al. *JAMA Network Open.*

eTable 2. Timeline of Vaccination Availability and Vaccine Guidelines Recommendations, December 2020 to December 2021

Date	Action
December 11, 2020	FDA approves the Pfizer mRNA COVID-19 vaccine
December 18, 2021	FDA approves Moderna mRNA COVID-19 vaccine
January 18, 2021	First date Pfizer recipients could be "fully vaccinated" (initial dose, second dose 21 days later, 14 days after 2nd dose)
January 22, 2021	First date Moderna recipients could be "fully vaccinated" (initial dose, second dose 28 days later, 14 days after 2nd dose)
February 27, 2021	FDA approves J&J COVID-19 vaccine
March 13, 2021	First date J&J recipients could be "fully vaccinated" (14 days after 1st dose)
August 13, 2021	CDC recommends that people with advanced HIV disease or unsuppressed HIV RNA and others with "severe immune suppression" conditions receive a third dose after 2 mRNA doses no less than 28 days after the second dose. There was not enough evidence to recommend additional doses for those vaccinated with J&J.<br>• Because our study participants were all fully vaccinated by June 30, 2021, our participants were ≥59 days since 2nd mRNA dose on August 13, 2021
September 24, 2021	CDC recommends Pfizer boosters for those ≥6 months after completing the Pfizer primary series among:<br>• ≥65 years of age and residents in long-term care settings<br>• 50-64 years of age with underlying medical conditions<br>CDC noted people 18-49 years with underlying conditions and people 18-64 who are at increased risk of COVID-19 exposure and transmission because of occupational or institutional settings *may* receive a booster
October 21, 2021	CDC recommends Pfizer or Moderna boosters for those ≥6 months after completing an mRNA primary series among:<br>• ≥65 years of age and residents in long-term care settings<br>• ≥18 years of age who live in long-term care settings<br>• ≥18 years of age who have underlying medical conditions<br>• ≥18 years of age who live or work in high-risk settings<br>CDC recommended booster shots for those ≥18 years of age who received J&J≥2 months ago.<br>CDC states individuals may choose their booster vaccine type (i.e., mix and match dosing for boosters)
November 19, 2021	CDC recommends boosters for all those ≥18 years of age who received an mRNA primary series ≥6 months ago
November 29, 2021	CDC strengthens recommendation from Nov 19, 2021, and expands to recommend all those ≥18 years of age who received J&J ≥2 months ago

© 2022 Coburn SB et al. *JAMA Network Open.*

eFigure 2. Distribution of Time From Fully Vaccinated to Receipt of Another Dose of Vaccine, by HIV Status, Among 133 994 Patients

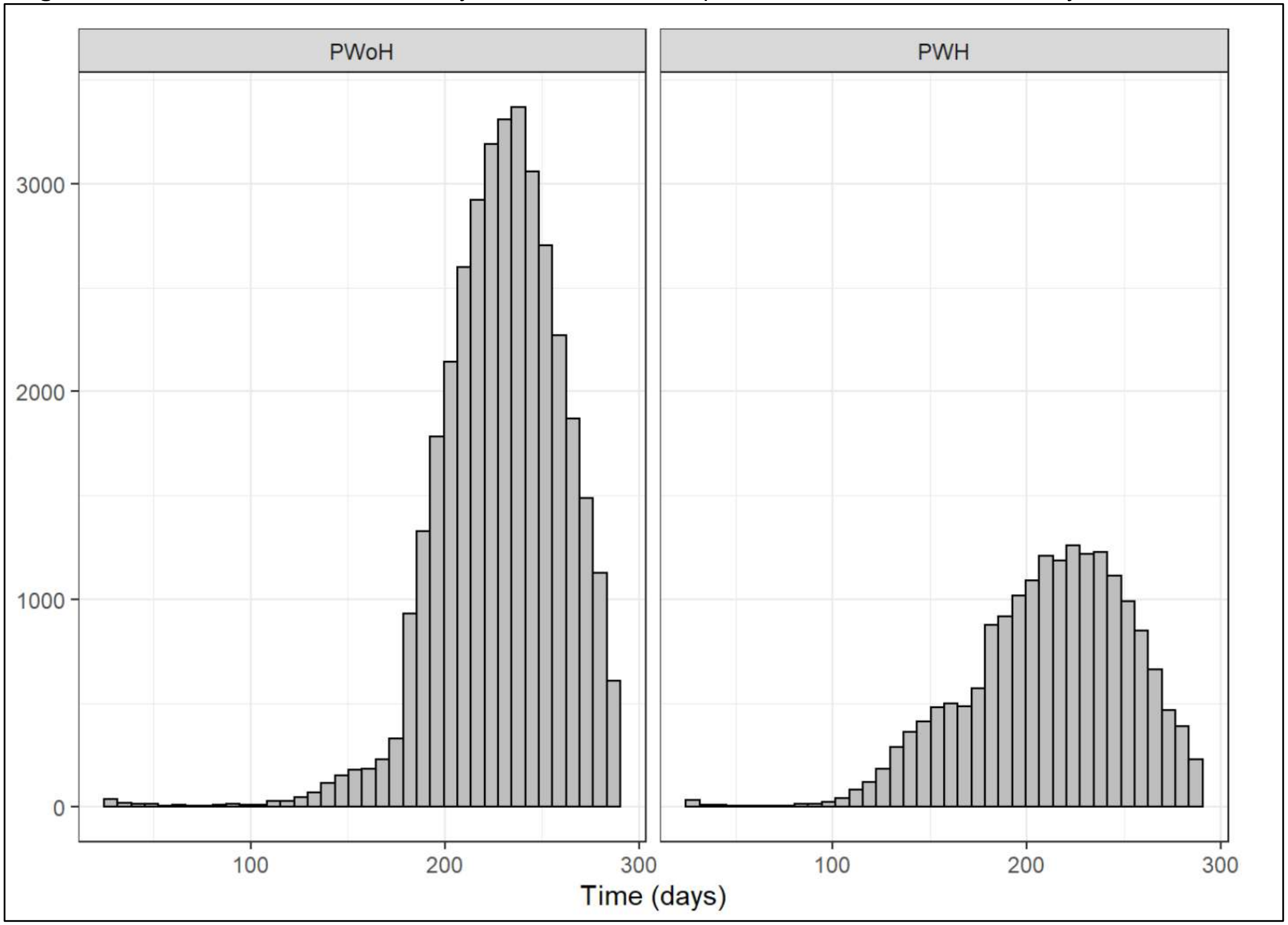

© 2022 Coburn SB et al. *JAMA Network Open.*

eTable 3. Characteristics of 33 029 PWH Who Did and Did Not Receive a Vaccine Dose After Their Primary Series

Characteristic	Overall N = 33,029[1]	Primary series only N = 14,644[1]	Primary series + dose < 5 months from full vaccination series N = 1,582[1]	Primary series + dose ≥ 5 months from full vaccination series N = 16,803	p-value[2]
**Age (years)**					<0.001
18-24	88 (0.3%)	62 (0.4%)	6 (0.4%)	20 (0.1%)	
25-34	1,614 (4.9%)	1,097 (7.5%)	71 (4.5%)	446 (2.7%)	
35-44	3,425 (10.4%)	1,920 (13.1%)	205 (13.0%)	1,300 (7.7%)	
45-54	5,766 (17.5%)	2,759 (18.8%)	389 (24.6%)	2,618 (15.6%)	
55-64	10,998 (33.3%)	4,646 (31.7%)	691 (43.7%)	5,661 (33.7%)	
65-74	8,575 (26.0%)	3,185 (21.7%)	193 (12.2%)	5,197 (30.9%)	
75+	2,563 (7.8%)	975 (6.7%)	27 (1.7%)	1,561 (9.3%)	
**Sex**					<0.001
Male	30,676 (92.9%)	13,482 (92.1%)	1,444 (91.3%)	15,750 (93.7%)	
Female	2,353 (7.1%)	1,162 (7.9%)	138 (8.7%)	1,053 (6.3%)	
**Ethnicity and Race**					<0.001
Hispanic	4,104 (12.4%)	1,782 (12.2%)	223 (14.1%)	2,099 (12.5%)	
Non-Hispanic Asian	1,034 (3.1%)	409 (2.8%)	77 (4.9%)	548 (3.3%)	
Non-Hispanic Black/ African American	13,545 (41.0%)	6,518 (44.5%)	505 (31.9%)	6,522 (38.8%)	
Non-Hispanic White	12,968 (39.3%)	5,293 (36.1%)	701 (44.3%)	6,974 (41.5%)	
Other	1,076 (3.3%)	493 (3.4%)	60 (3.8%)	523 (3.1%)	
Unknown	302 (0.9%)	149 (1.0%)	16 (1.0%)	137 (0.8%)	
**Month fully vaccinated**					<0.001
January 2021	359 (1.1%)	164 (1.1%)	3 (0.2%)	192 (1.1%)	
February 2021	3,801 (11.5%)	1,641 (11.2%)	25 (1.6%)	2,135 (12.7%)	

© 2022 Coburn SB et al. *JAMA Network Open.*

Characteristic	Overall<br>N = 33,029[1]	Primary series only<br>N = 14,644[1]	Primary series + dose < 5 months from full vaccination series<br>N = 1,582[1]	Primary series + dose ≥ 5 months from full vaccination series<br>N = 16,803	p-value[2]
March 2021	8,702 (26.3%)	3,173 (21.7%)	48 (3.0%)	5,481 (32.6%)	
April 2021	12,306 (37.3%)	5,001 (34.2%)	768 (48.5%)	6,537 (38.9%)	
May 2021	5,793 (17.5%)	3,151 (21.5%)	604 (38.2%)	2,038 (12.1%)	
June 2021	2,068 (6.3%)	1,514 (10.3%)	134 (8.5%)	420 (2.5%)	
**Primary vaccination series**					<0.001
Pfizer	17,389 (52.6%)	7,296 (49.8%)	885 (55.9%)	9,208 (54.8%)	
Moderna	13,629 (41.3%)	6,080 (41.5%)	660 (41.7%)	6,889 (41.0%)	
J&J	2,011 (6.1%)	1,268 (8.7%)	37 (2.3%)	706 (4.2%)	
**COVID prior to fully vaccinated**	2,272 (6.9%)	1,086 (7.4%)	120 (7.6%)	1,066 (6.3%)	<0.001
**CD4 at ART initiation**	368.00 (202.00, 584.00)	373.00 (207.00, 594.00)	356.00 (182.00, 555.00)	364.00 (198.00, 580.00)	<0.001
Unknown	14,910 (45.1%)	6,275 (42.9%)	775 (49.0%)	7,860 (46.8%)	
**AIDS before fully vaccinated**	8,335 (25.2%)	3,543 (24.2%)	343 (21.7%)	4,449 (26.5%)	0.008
**CD4 at fully vaccinated**	636 (449, 858)	634 (443, 858)	632 (430, 849)	640 (455, 859)	0.070
Unknown	6,393 (19.3%)	3,244 (22.2%)	204 (12.9%)	2,945 (17.5%)	
**Suppressed (<50 copies/mL) HIV RNA at fully vaccinated**	26,052 (90.5%)	10,810 (88.8%)	1,345 (90.5%)	13,897 (91.9%)	<0.001
Unknown	4,240 (12.8%)	2,466 (16.8%)	95 (6.0%)	1,679 (10.0%)	

[1]n (%); Median (IQR)

[2]Pearson's Chi-squared test; Wilcoxon rank sum test

© 2022 Coburn SB et al. *JAMA Network Open.*

eTable 4. Breakthrough Infection Incidence Rates and 95% CIs, by Vaccine Type and HIV Status

		# of breakthroughs	# of person-years	IR (95% CI) per 1,000 person years
**Moderna**				
	Overall	1,140	33,262.7	34.3 (32.3, 36.3)
	PWH	369	9,391.2	39.3 (35.4, 43.5)
	PWoH	771	23,871.5	32.3 (30.1, 34.7)
**Pfizer**				
	Overall	2,158	40,017.5	53.9 (51.7, 56.3)
	PWH	770	11,909.0	64.7 (60.2, 69.4)
	PWoH	1,388	28,108.5	49.4 (46.8, 52.1)
**J&J**				
	Overall	351	4999.5	70.2 (63.1, 78.0)
	PWH	102	1,327.9	76.8 (62.6, 93.3)
	PWoH	249	3,671.6	67.8 (59.7, 76.8)
**By calendar month**				
Jan	Overall	1	25.0	(1.0, 222.6)
	PWH	0	7.5	0.0 (0.0, 492.3)
	PWoH	1	17.5	57.0 (1.4, 317.8)
Feb	Overall	13	445.7	29.2 (15.5, 49.9)
	PWH	6	139.5	43.0 (15.8, 93.6)
	PWoH	7	306.2	22.9 (9.2, 47.1)
Mar	Overall	52	2322.9	22.4 (16.7, 29.4)
	PWH	17	704.3	24.1 (14.1, 38.6)
	PWoH	35	1618.6	21.6 (15.1, 30.1)
Apr	Overall	107	5426.0	20.4 (16.7, 24.6)
	PWH	40	1551.1	25.8 (18.4, 35.1)
	PWoH	67	3694.9	18.1 (14.1, 23.0)
May	Overall	95	8338.5	11.4 (9.2, 139)
	PWH	28	2418.1	11.6 (7.7, 16.7)
	PWoH	67	5920.4	11.3 (8.8, 14.4)
June	Overall	113	9082.6	12.4 (10.2, 15.0)
	PWH	43	2624.6	16.4 (11.9, 22.1)
	PWoH	70	6458.0	10.8 (8.4, 13.7)
Jul	Overall	296	9557.6	72.1 (66.8, 77.8)
	PWH	136	2764.2	49.2 (41.3, 58.2)
	PWoH	160	6793.5	23.6 (20.0, 27.5)
	Overall	684	9481.8	72.1 (66.8,77.8)

© 2022 Coburn SB et al. *JAMA Network Open.*

Aug	PWH	259	2734.7	94.7 (83.5, 107.0)
	PWoH	425	6747.1	63.0 (57.1, 69.3)
Sept	Overall	497	9096.7	54.6 (49.9,59.7)
	PWH	162	2621.2	61.8 (52.6, 72.1)
	PWoH	335	6475.5	51.7 (46.3, 57.6)
Oct	Overall	294	9320.8	31.5 (28.0, 35.4)
	PWH	82	2683.4	30.6 (24.3, 37.9)
	PWoH	212	6637.3	31.9 (27.8, 36.5)
Nov	Overall	232	8590.3	27.0(23.6, 30.7)
	PWH	81	2463.1	32.9 (26.1, 40.9)
	PWoH	151	6127.2	24.6 (20.9, 28.9)
Dec	Overall	1,265	7087.5	178.5 (168.8, 188.6)
	PWH	387	2008.0	192.7 (174.0, 212.9)
	PWoH	878	5079.6	172.8 (161.6, 184.7)

© 2022 Coburn SB et al. *JAMA Network Open.*
